# Assessment of changes in the national surveillance data for adult and pediatric VAE during the COVID-19 pandemic in hospitals

**DOI:** 10.1017/ash.2023.406

**Published:** 2023-09-29

**Authors:** Vaishnavi Pattabiraman, Jennifer Watkins, Sheila Abner, Lindsey Lastinger

## Abstract

**Background:** Among US acute-care hospitals (ACHs) reporting to the NHSN, significant increases in the incidence of Ventilator-Associated Events (VAEs) were observed during the COVID-19 pandemic years in comparison with 2019. We assessed changes in the national event-level VAE data, including the incidence of specific event-types: Ventilator-Associated Condition (VAC), Infection-related Ventilator-Associated Complication (IVAC) and Possible Ventilator-Associated Pneumonia (PVAP). We also examined changes in associated pathogens, and we evaluated incidence density rates (IDRs) of pediatric VAE (PedVAE) before and during the pandemic years. **Methods:** We analyzed data on VAE and PedVAE reported to NHSN between 2019 through the second quarter of 2022 (2022Q2) in ACHs. Annual proportions of VAC, IVAC, or PVAP were calculated; changes versus 2019 were assessed. The 10 most common PVAP pathogens reported annually were examined, and the percentages and ranks for each were calculated. Among pediatric and neonatal locations, PedVAE IDR were calculated as the number of events per 1,000 ventilator days and were compared between the pre-pandemic and pandemic years. All comparisons were conducted using a mid-P exact test, and P<0.05 was considered statistically significant. **Results:** Between 1,266 - 1,357 ACHs reported VAE data each year. A total of 24,836 (2019), 37,592 (2020), and 50,362 (2021) VAEs were reported. The proportion of VAC events in 2020 (64.1%) was significantly higher than in 2019 (62.9%), while the 2020 and 2021 PVAP proportions (8.7% and 9.2%, respectively) were significantly lower than in 2019 (10.0%). The majority of VAEs were reported from the same location types annually. The top 3 PVAP pathogens reported for each year remained unchanged: Staphylococcus aureus, Pseudomonas aeruginosa, Klebsiella. However, the proportion identified as Haemophilus influenzae decreased significantly each year from 2019-2021, with the rank dropping from #5 in 2019 (6.6%) to #10 in 2021 (2.3%). Between 199 – 257 ACHs conducted PedVAE surveillance. PedVAE IDR were significantly lower in 2020 (0.8), 2021 (1.1), and the first half of 2022 (0.8) when compared to 2019 (1.3). **Conclusions:** This study provides a national view of specific VAEs before and during the COVID-19 pandemic. Some changes in the associated pathogens, and the proportions of VAC and PVAP, were observed. This study is the first to produce national benchmarks for PedVAE IDR. Additional ACHs conducting PedVAE surveillance in NHSN would improve the representativeness of our results.

**Disclosures:** None

